# Genetic Determinants and Breeding Strategies for Rice Cooking and Eating Quality: A Comprehensive Review

**DOI:** 10.1111/pbi.70447

**Published:** 2025-11-04

**Authors:** Guangming Lou, Pei Fu, Haozhou Gao, Minqi Li, Huan Shi, Rongjia Liu, Yanhua Li, Hao Zhou, Duo Xia, Yuexing Wang, Guanjun Gao, Yuqing He

**Affiliations:** ^1^ National Key Laboratory of Crop Genetic Improvement and National Center of Plant Gene Research (Wuhan), Hubei Hongshan Laboratory Huazhong Agricultural University Wuhan China; ^2^ State Key Laboratory of Rice Biology and Breeding China National Rice Research Institute Hangzhou China; ^3^ Institute of Food Crop Hubei Academy of Agricultural Science Wuhan China

**Keywords:** breeding strategies, cooking and eating quality, starch, protein, lipid, rice

## Abstract

Rice cooking and eating quality (CEQ), a core agronomic trait tied to consumer preference and market value, is regulated by endosperm starch, protein, and lipid metabolism; this review synthesizes advances in its molecular mechanisms, focusing on genetic determinants and regulatory networks of storage substances. Starch is key: the *Wx* gene (central hub) has ≥ 9 allelic variants (e.g., *Wx*ᵃ, *Wx*ᵇ and *Wx* ^ela^), modulating amylose content (AC) via GBSSI activity, *Wx* gene expression levels, splicing efficiency, etc., and exerting pleiotropic effects on protein/lipid metabolism, grain transparency, and taste; storage proteins (80% glutelin) harm CEQ by inhibiting starch gelatinisation, regulated by relevant gene families and vesicular pathways (e.g., Rab5a‐VPS9a); lipids (0.3%–3% of endosperm) affect Rapid Visco Analyzer (RVA) profiles and aroma via genes like *OsFAD*, *PDCT*, *FGC3*; modules (e.g., OsNF‐Ys, OsbZIP60, Ghd7‐OsNAC42) balance starch‐protein accumulation. Breeding strategies include marker‐assisted selection, CRISPR/Cas9 editing of *Wx* elements or glutelin genes, gene pyramiding (*Wx*, *ALK*, *GS3*), and artificial intelligence (AI)‐integrated intelligent breeding to boost efficiency; the review first proposes a framework: ‘*Wx* genotype determination – amylopectin/glutelin regulation – CEQ improvement’, and the establishment of this framework is rooted in the core role of the *Wx* gene in determining rice eating quality, which is achieved through its pleiotropic regulation of starch, protein, and lipid metabolism in the endosperm. Future directions: use *Wx* allelic diversity, decipher multi‐omics modules (e.g., RNA methylation in starch metabolism), and integrate aroma, Nitrogen Use Efficiency (NUE), grain shape, chalkiness, sugar transport genes to balance yield and CEQ for rice breeding.

## 1 Introduction

The importance of tiny rice grains in human daily lives cannot be overstated; after all, half of the world's population relies on rice for daily energy and nutrition (Bao et al. 2023). Since the 1960s, a substantial increase in rice yield has been achieved through semi‐dwarf breeding, extensive use of nitrogen fertiliser, utilisations of hybrid advantage, and super rice breeding. To some extent, this has alleviated the pressure of food shortage caused by the population explosion and insufficient arable land area. With the gradual satisfaction of people's subsistence and the pursuit of a quality lifestyle, high‐quality rice is increasingly favoured by consumers. Therefore, the improvement of rice quality has become one of the top priorities in modern rice breeding programs.

Rice quality encompasses the characteristics of rice as a market commodity, reflecting both its physical and chemical properties. It can be categorised into several types: appearance quality (AQ), cooking and eating quality (CEQ), nutritional quality (NQ), milling quality (MQ), and recently—recognised hygiene quality (HQ) (Zhou et al. 2019). AQ typically encompasses grain shape, which includes parameters such as grain length, width, thickness, and the length‐to‐width ratio, as well as chalkiness (the opaque region within the grain endosperm) and transparency. CEQ primarily reflects the properties and palatability of cooked rice. The NQ of rice is affected by the quantity and quality of starch, protein, vitamins, minerals, and other chemical components within the grains that are beneficial to human health. MQ refers to the integrity of rice during processing, and it is commonly measured by brown rice yield, milled rice yield, and head rice yield. HQ of rice mirrors the extent of both internal and external contamination. Its assessment typically involves the detection of toxic heavy metals, pesticide residues, and allergens.

While rice quality is a crucial determinant of market price and consumer preference, rice CEQ is the most significant factor influencing its price (Bao et al. 2023). As the most critical quality trait, rice CEQ has diversified evaluation indexes. Generally, rice CEQ is measured by sensory evaluations such as the appearance, viscosity, hardness, palatability, and the smell of rice. In recent years, rice taste value has also been used as a criterion for evaluating rice CEQ. For instance, the rice taste analyzer manufactured by Japan's Satake Corporation can calculate physical parameters such as viscosity, elasticity, hardness and balance by measuring the optical characteristics of rice, thus calculating the predicted taste value (Ohtsubo and Nakamura 2017). Moreover, rice CEQ can also be quantitatively analyzed by determining physical and chemical indicators such as the starch RVA spectral characteristics (e.g., gelatinisation temperature, peak viscosity, hot paste viscosity, final viscosity, breakdown value, and setback value), amylose content, and gel consistency (Shi et al. 2022).

**FIGURE 1:**
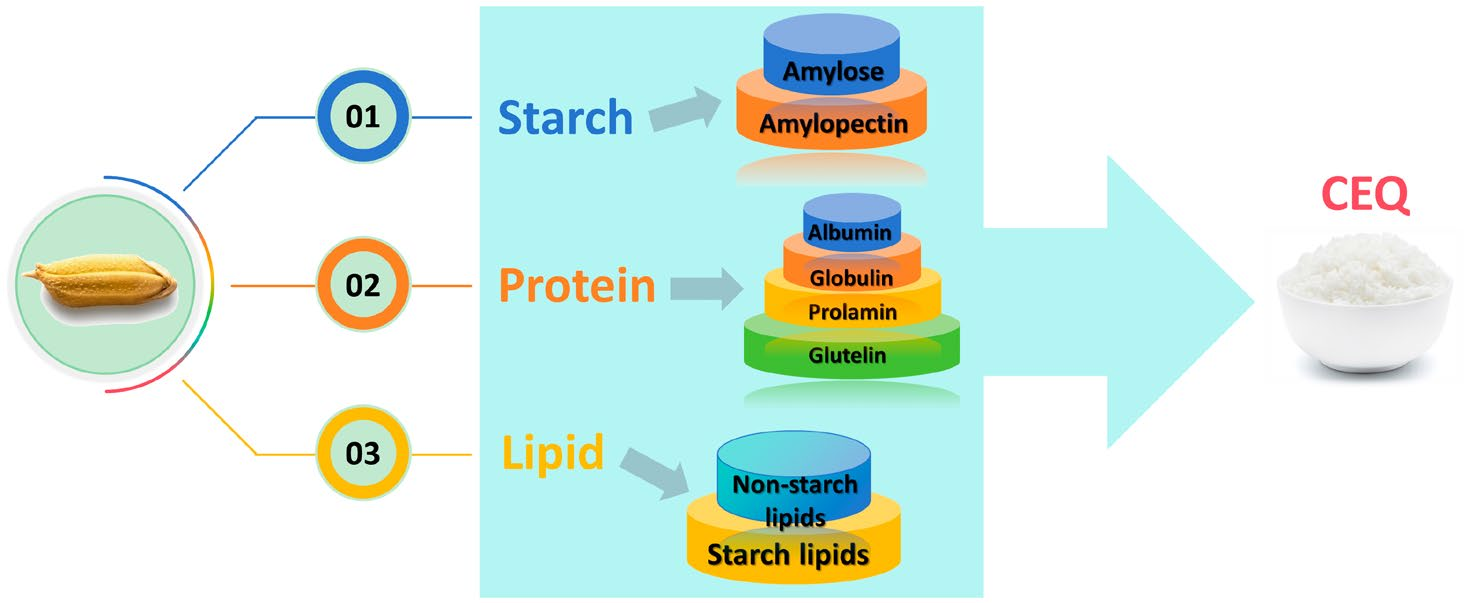
The cooking and eating quality of rice is jointly determined by three major components of rice endosperm: starch, protein, and lipid.

Although in recent years some scientists have begun to advocate the consumption of whole grains, which are believed to provide more nutrients (Zhang 2021), refined rice (the endosperm that remains after removing the seed coat, the aleurone layer and the embryo) is still the main form of human consumption. Therefore, the synthesis, composition, distribution, and accumulation of the endosperm's three major components—starch, protein, and lipid—determine the CEQ of rice (Figure 1).

**FIGURE 2:**
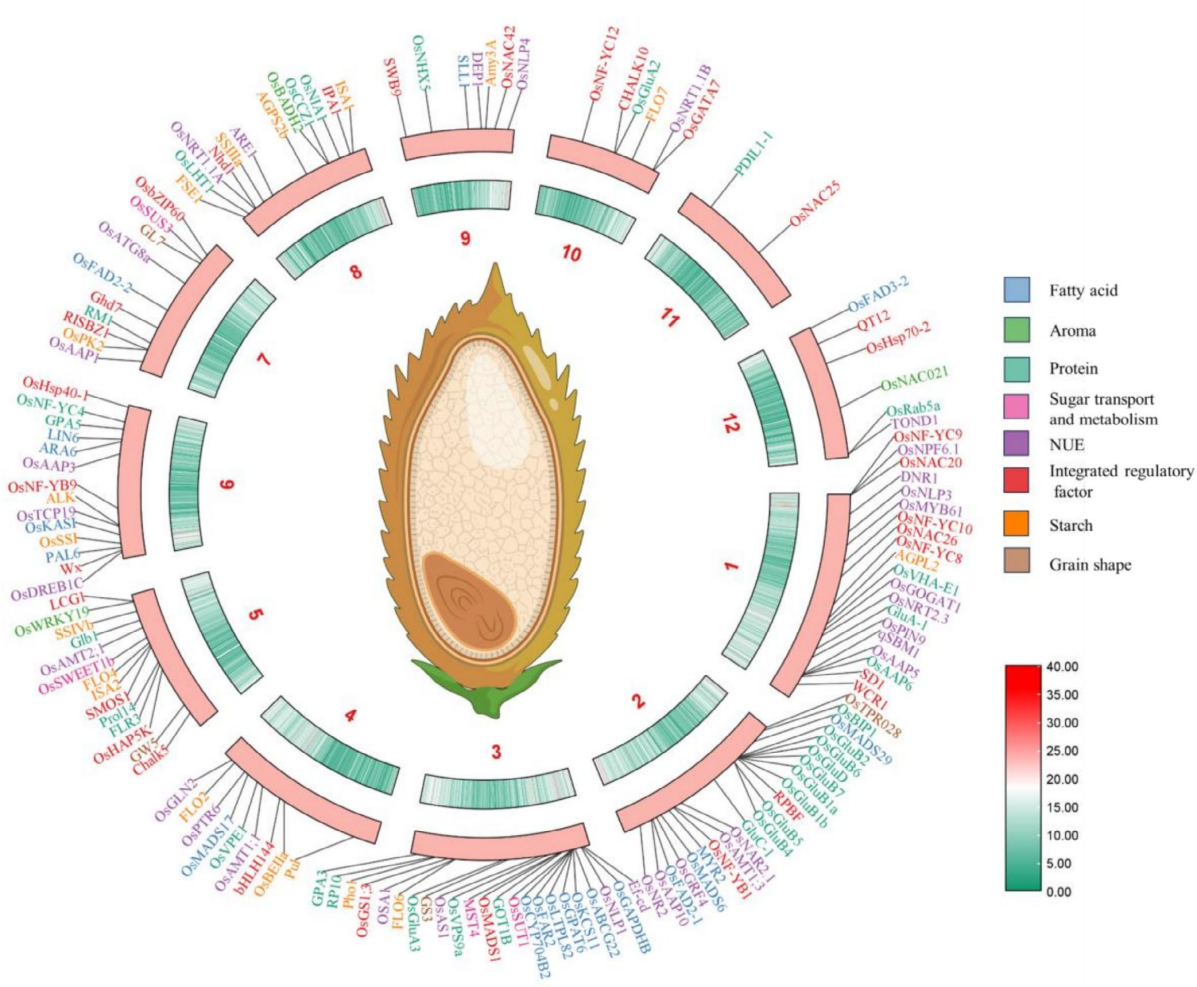
Distribution map of confirmed and potential CEQ genes on 12 chromosomes. The outermost circle represents chromosomes, and the innermost circle presents gene density in a heat map. All key information of the CEQ‐related genes in the figure (including gene ID, gene name, specific effects on rice cooking and eating quality, and references) can be found in Table S2.

In this review, we summarise recent advances in the genetic regulation of rice CEQ, focusing on molecular mechanisms governing starch, protein, and lipid metabolism—with emphasis on the central role of the *Wx* gene. We also compile key genes validated or potentially involved in CEQ regulation (Figure 2) and outline breeding strategies for improving rice quality. This synthesis aims to support the efficient development of ideal rice varieties.

## 2 Key Factors and Genetic Basis for Rice CEQ Improvement

### 2.1 Starch——The Most Important Determinant of Rice CEQ

#### 2.1.1 Amylose

##### 2.1.1.1 The Composition of Rice Starch and the Abundant Natural Variations of the **Wx** Gene

Rice grains are composed of approximately 85%–90% starch, 7%–12% protein, and 0.3%–3% lipid (Zhou et al. 2002). The starch in rice is primarily categorised into two types: linear amylose (composed of glucose units linked by α‐1,4‐glycosidic bonds) and branched amylopectin (composed of α‐1,4‐glycosidic bonds in linear chains with α‐1,6‐glycosidic bonds at branch points). Amylose typically constitutes 15%–25% of rice starch, while amylopectin accounts for 75%–85%. Consistent studies indicate that the amylose content (AC) in the rice endosperm is the most critical factor determining the physicochemical properties and final quality of rice (Li and Gilbert 2018). Therefore, the main gene *Wx* responsible for amylose synthesis in rice is the most important genetic factor to determine the cooking and eating quality of rice.

**FIGURE 3:**
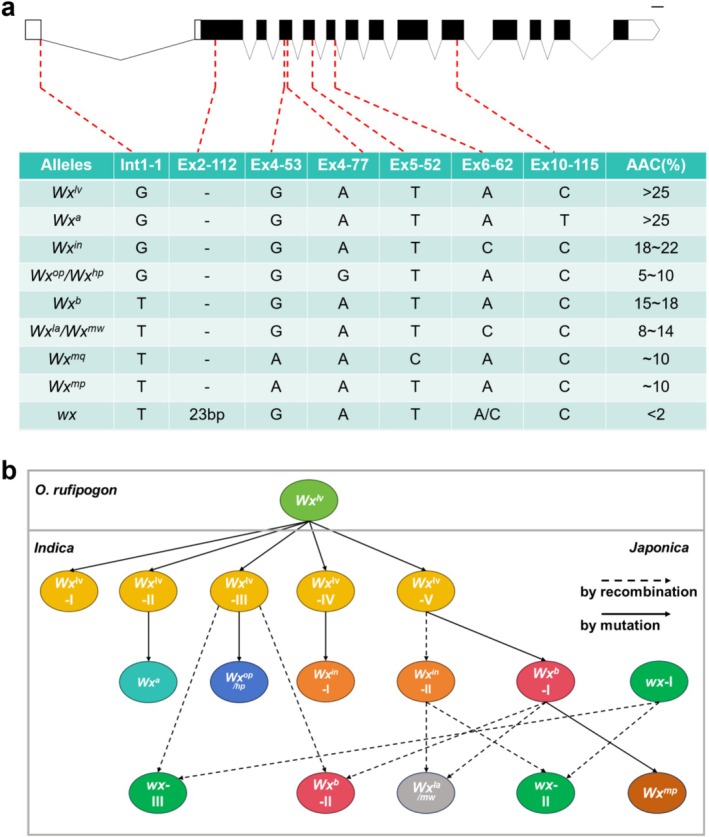
Genetic diversity and evolutionary relationship among multiple *Wx* alleles in rice. (a) Genotypes of different *Wx* alleles. The upper part of the figure shows the genomic structure of the *Wx* gene, while the lower part illustrates the nucleotide variations of each allele at seven functional polymorphic sites. (b) Proposed evolutionary relationship among various *Wx* alleles in rice. *Wx* ^*lv*^‐I, *Wx* ^*lv*^‐II, *Wx* ^*lv*^‐III, *Wx* ^*lv*^‐IV and *Wx* ^*lv*^‐V indicate the five haplotypes of *Wx* ^*lv*^ alleles in *O. sativa* . *Wx* ^*in*^‐I, *Wx* ^*in*^‐II indicate the two haplotypes of *Wx* ^*in*^ alleles in *O. sativa* . *wx*‐I, *wx*‐II and *wx*‐III indicate the three haplotypes of *wx* alleles in *O. sativa* . The results regarding the classification of *Wx* gene haplotypes and their evolutionary relationships presented in this figure are based on previous studies (Zhang et al. 2020; Zhang, Zhu, et al. 2020; Zhou, Xia, Zhao, et al. 2021).

The genetic diversity of the rice variety AC is intricately associated with the allelic variations within the *Wx* gene. To date, a minimum of nine natural allelic variants of the *Wx* gene have been identified, namely *wx*, *Wx* ^*a*^, *Wx* ^*in*^, *Wx* ^*b*^, *Wx* ^*la*^/*Wx* ^*mw*^, *Wx* ^*mp*^, *Wx* ^*mq*^, *Wx* ^*op*^/*Wx* ^*hp*^ and *Wx* ^*lv*^ (Figure 3a). Among these, the non‐waxy gene (*Wx*) exhibits incomplete dominance over the waxy gene (*wx*). *Wx* ^*a*^ is predominantly distributed in indica rice, and rice plants carrying this allele have high AC in grain, often above 25%; *Wx* ^*b*^ is predominantly distributed in japonica rice, with intermediate or low grain AC (15%–18%). The single‐nucleotide variation (Int1‐1) at the 5′ splice site of the first intron in *Wx* ^*a*^ and *Wx* ^*b*^ alleles leads to significant differences in the post‐transcriptional level of *Wx* and the final AC in grains between indica and japonica rice (Hirano et al. 1998). Previous association analyses of diverse rice collections demonstrated that base variants at the Ex10‐115 locus of the *Wx* gene were associated with soft gel consistency (GC) and low RVA phenotypes (Chen et al. 2008). In the *Wx* ^*lv*^ allele, the G nucleotide in Int1‐1 and the C nucleotide in Ex10‐115 are the single nucleotide polymorphisms (SNPs) that lead to high apparent amylose content (AAC) and low viscosity (Zhang, et al. 2020). Except for *Wx* ^*a*^ with a T nucleotide, all other *Wx* alleles, most wild rice accessions, and the orthologs encoding GBSSI from all the tested monocotyledons [maize ( *Zea mays* ), sorghum ( *Sorghum bicolor* ), millets (*Setaria virdis*, *Setaria italica* ), *Panicum Hallii* , *Panicum virgatum* , *Brachypodium stacei*, and *Brachypodium distachyon* ] had C at the Ex10–115 site, indicating that the C nucleotide in Ex10‐115 is ancestral. Compared with *Wx* ^a^ and *Wx* ^b^, the nucleotides at Int1‐1 and Ex6‐62 positions in *Wx* ^in^ are G and C, respectively, resulting in a reduction of the encoded mature mRNA and a decrease in GBSSI protein levels (Mikami et al. 2008). The *Wx* ^*op*^/*Wx* ^*hp*^ allele, which carries an A‐to‐G change at Ex4‐77 position resulting in an Asp165/Gly165 substitution, had no detectable effects on GBSSI activity in vitro but notably reduced the binding of GBSSI to starch granules, thereby leading to a reduction of AC in rice seeds (Liu et al. 2009). Derived from the mutation of *Wx* ^b^, the *Wx* ^mq^ allele contains two position mutations at Ex4–53 and Ex5–52, which do not affect the gene's expression level yet result in a reduction in GBSSI enzyme activity (Sato et al. 2002). *Wx* ^mp^ is formed by reverse mutation of *Wx* ^mq^ and contains only the mutated Ex5–52 site (Yang et al. 2013). Rice with low amylose content is favoured by consumers in many regions due to its generally superior CEQ and acceptability (Calingacion et al. 2014). However, rice varieties with low amylose content often exhibit a dull or opaque grain appearance, which diminishes their commercial value due to this unattractive visual quality (Zhou, Xia, Zhao, et al. 2021). The discovery of the *Wx* ^la^/*Wx* ^mw^ alleles has perfectly resolved this issue, as rice carrying the *Wx* ^la^/*Wx* ^mw^ alleles possesses low amylose content and transparent grain appearance (Zhang, Yang, et al. 2020; Zhou, Xia, Zhao, et al. 2021). The *Wx* ^la^/*Wx* ^mw^ alleles are generated through intragenic recombination of *Wx* ^b^ and *Wx* ^in^ (Zhou, Xia, Zhao, et al. 2021). Recently, a novel *Wx* allele, *Wx* ^ela^, has been identified. This allele arises from a C to T mutation at the 20th base of the 12th exon of *Wx*, which affects the GT1 domain of GBSSI, thereby reducing GBSSI activity and the amylose content in grains (Lin et al. 2024).

##### 2.1.1.2 Evolutionary Relationships of the *Wx* Gene

Based on the gene sequence information and regional distribution characteristics of different genotypes, preliminary evolutionary relationships among various *Wx* gene allelic variations have been proposed (Figure 3b). The gene sequence of *Wx* ^lv^ is similar to that of most wild rice, and it is considered the ancestral allele genotype in cultivated rice varieties. The subspecies or subgroups of modern cultivated rice originated from multiple wild rice populations. The *Wx* ^lv^ allele has diverged into multiple haplotypes due to artificial selection and domestication. During the early domestication of rice, a functional SNP mutation occurred at Int1‐1 or Ex6‐62, leading to the evolution of the *Wx* ^lv^ allele into *Wx* ^b^ and *Wx* ^in^, respectively. Meanwhile, the *Wx* ^lv^ gene haplotypes in different regions mutate into *Wx* ^a^ and *Wx* ^hp^/*Wx* ^op^ due to base mutations at the Ex10‐115 or Ex4‐77 sites, respectively. *Wx* ^b^ and *Wx* ^in^ are consistently found in the *japonica* subspecies, and during the subsequent cultivation of *japonica* rice, *Wx* ^b^ is selected and further domesticated, resulting in the emergence of the non‐functional *wx* allele caused by the insertion of a 23 bp fragment at the Ex2‐113 site. In fact, the recombination rate at the *Wx* locus was estimated to be 3.34 kb/cM, approximately 75 times higher than the genome‐wide average, indicating that intragenic recombination serves as the primary driving force behind the diversity of the *Wx* locus (Zhou et al. 2021). Novel *Wx* genotypes can be easily generated by crossing different *Wx* genotypes (Zhou et al. 2021). Notably, the proportion of variation sites (9.76%) and the polymorphism rate (*π* = 0.0936) of the *Wx* gene in wild rice are significantly higher than those observed in cultivated rice (Li 2015). This finding suggests that wild rice resources may exhibit a broader diversity in AC compared to their cultivated counterparts.

##### 2.1.1.3 The *Wx* Gene Exhibits Strong Pleiotropy

While the role of the *Wx* gene in regulating grain starch content is well established, its contributions to CEQ extend far beyond this function. Zhou et al. introduced the *waxy* gene region (*Wx* ^b^) from Minghui 63 into Zhenshan 97 (*Wx* ^a^), resulting in improved lines (Zhenshan 97B/Zhenshan 97A) and their hybrid combinations with Minghui 63. These derivatives exhibited reduced amylose content, increased gel consistency, adjusted gelatinisation temperature, and enhanced grain transparency, thus achieving synchronous improvement in amylose content, gel consistency, gelatinisation temperature, and grain opacity (Zhou et al. 2003). Tan et al. (2001) performed genetic mapping of milling traits, protein content, and flour colour using recombinant inbred lines (RILs) derived from the widely cultivated hybrid rice combination ‘Shanyou 63’ in China and showed that the *Wx* gene played a dominant role in determining protein content and flour colour with minor modifications from several polygenic QTLs. Wang et al. (2007) analyzed the genetic basis of 17 traits (or parameters) characterising rice cooking and eating quality using a RIL population derived from a cross between Zhenshan 97 and Delong 208 and identified a QTL cluster corresponding to the *Wx* locus that simultaneously regulated AC, GC, alkali spreading value (ASV), and most viscosity parameters with no effect on peak viscosity (PKV) or consistency (CRE). Xia et al. (2022) investigated the quality performance of different *Wx* genotypes under varying nitrogen fertilisation levels and temperature conditions during grain development and found that eating quality deteriorated with enhanced *Wx* gene function while the *Wx* gene also regulated starch‐lipid complex content. Ge et al. (2005) conducted QTL analysis using a RIL population from the indica varieties ‘Zhenshan 97’ and ‘Minghui 63’—the most widely planted hybrid rice parents in China—and identified a single QTL near the *Wx* gene on chromosome 6 that influenced all tested rice quality traits. Chen et al.'s (2018) genome‐wide association study (GWAS) revealed significant associations between rice grain protein content (including four storage protein fractions) and the *Wx* gene region, with a particularly inverse correlation between the 2.3 kb mRNA level of *Wx* and albumin content. Xia et al. (2022) discovered in genome‐wide association studies that the *Wx* gene acts as a negative regulator for both crude fatty acid content and rice quality. Studies by Zhou et al. established the *Wx* gene as a major determinant of variation in grain transparency and eating quality (Zhou et al. 2021). Collectively, these results indicate that the *Wx* gene comprehensively regulates amylose content, protein content, lipid content, taste value, and nearly all parameters used to characterise the physicochemical properties of starch.

**FIGURE 4:**
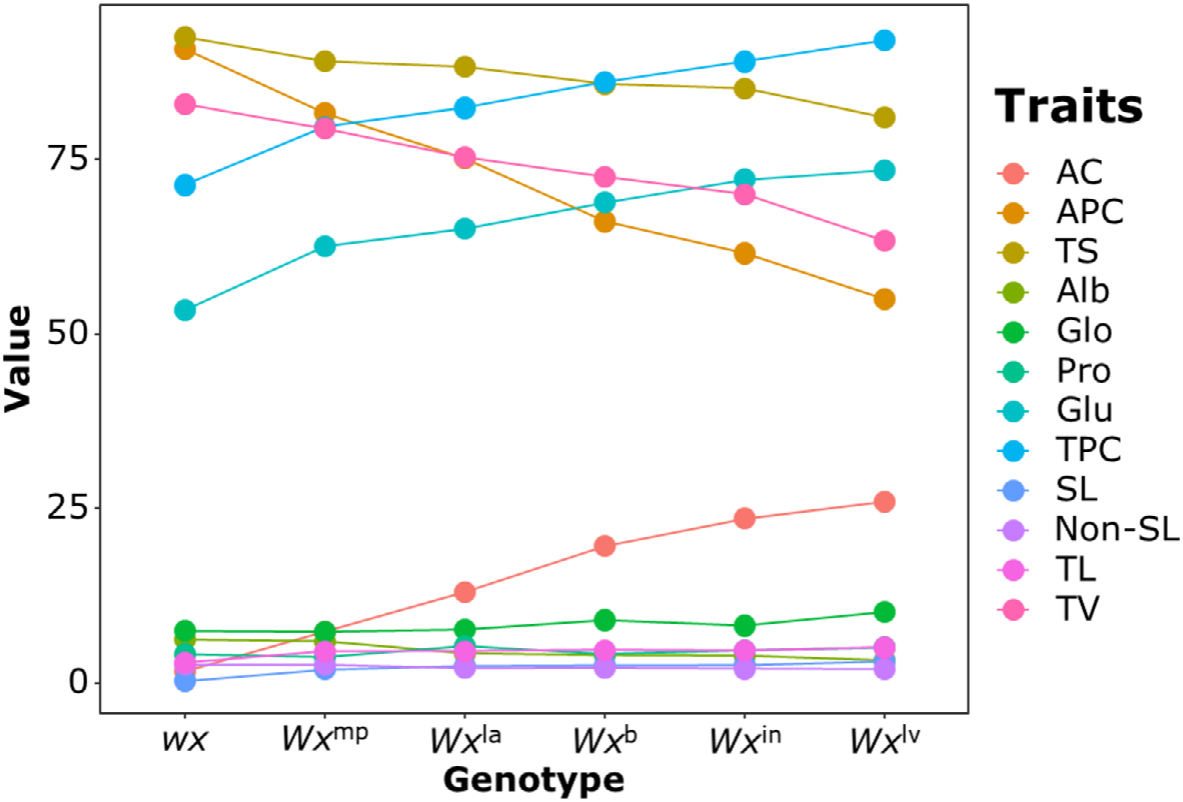
Effects of different *Wx* allelic genotypes on various rice quality traits. All data are presented as means (*n* ≥ 12). The error bars are not shown in the figure because their labeling is not conducive to observing the variation trends. The detailed data and results of one‐way ANOVA can be found in Table S1. AC, amylose content; Alb, albumin; APC, amylopectin content; Glo, globulin; Glu, glutelin; Non‐SL, non‐starch lipid; Pro, prolamin; SL, starch‐lipid; TL, total lipid; TPC, total protein content; TS, total starch; TV, taste value.

To fully validate the pleiotropic functions of the *Wx* gene, we conducted comprehensive analyses of starch, protein, lipid, and taste values using a set of transgenic materials in the Zhennuo genetic background carrying different *Wx* alleles (Figure 4). The results showed that as the functional intensity of the *wx*, *Wx* ^mp^, *Wx* ^la^, *Wx* ^b^, *Wx* ^in^, and *Wx* ^lv^ alleles increased sequentially, in addition to the gradual increase in amylose content, both amylopectin and total starch contents decreased progressively, showing an opposite trend to that of amylose. Among the four storage protein fractions, except for albumin, which decreased with the increase in amylose, globulin, prolamin, glutelin, and total protein all increased as amylose content rose. Non‐starch lipids exhibited a trend opposite to that of amylose, while starch lipids and total lipids increased with the elevation of amylose. The taste value increased with the increase in amylopectin content. Since the edible part of rice is mainly the endosperm, whose primary components are starch, protein, and lipid, and the *Wx* gene pleiotropically determines the traits of starch, protein, and lipid, *Wx* is thus the key gene governing rice CEQ.

**FIGURE 5:**
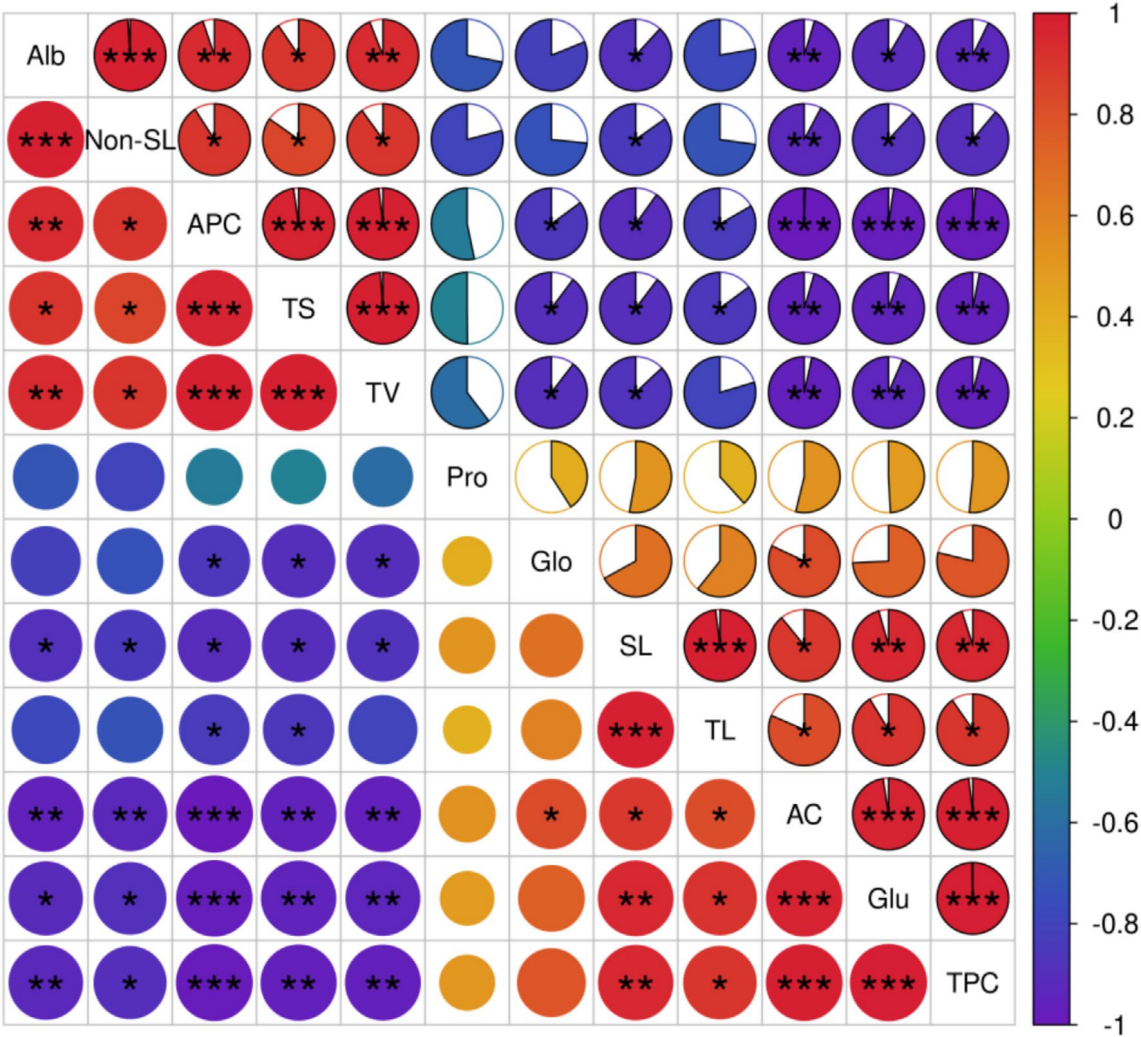
Heatmap of correlation analysis among various quality traits. AC, amylose content; Alb, Albumin; APC, amylopectin content; Glo, globulin; Glu, glutelin; Non‐SL, Non‐starch lipid; Pro, prolamin; SL, starch‐lipid; TL, total lipid; TPC, total protein content; TS, total starch; TV, taste value. Significant differences were determined using Pearson correlation analysis. The colour gradient represents the correlation coefficient (ranging from −1 to 1), with red indicating a positive correlation and blue indicating a negative correlation; *, **, and *** denote significant correlations at the *p* < 0.05, *p* < 0.01, and *p* < 0.001 levels, respectively.

How the composition of endosperm starch, protein, and lipid affects eating quality? Further analysis of Figure 5 revealed that amylose content, glutelin content, and total protein content exhibited a highly significant negative correlation with taste value; given that glutelin is the main component of wheat gluten—a factor influencing noodle hardness—it similarly affects the hardness of cooked rice, and as amylose content increases, cooked rice becomes increasingly fluffy and firm, thus the impact of amylose on eating quality is primarily mediated by glutelin. In contrast, amylopectin content and total starch content showed a highly significant positive correlation with taste value, and as amylopectin content rises, cooked rice becomes progressively more sticky (Figure 4), indicating that amylopectin content mainly influences the stickiness of cooked rice; additionally, non‐starch lipids were found to have a significant positive correlation with rice taste value, and notably, glutinous rice carrying the *wx* allele has the highest non‐starch lipid content, which primarily contributes to the lustre and aroma of cooked rice. In rice endosperm, glutelin accounts for approximately 80% of the total protein content, while amylopectin constitutes 70%–100% of the total starch content, making them the dominant components of endosperm protein and starch, respectively; therefore, the effect of the *Wx* gene on rice eating quality is primarily the result of the combined actions of amylopectin content, glutelin content, non‐starch lipids, and other related components.

##### 2.1.1.4 *Wx* Determines the Dietary Preferences of Different Populations

Regional dietary preferences have shaped the distribution of *Wx* alleles through artificial selection. *Indica* rice varieties carrying the *Wx* ^a^ allele have high amylose content, resulting in rice with a harder and looser texture, which is suitable for dry cooking methods such as fried rice and is popular among people in Southeast Asia. In contrast, Northeast Asia is dominated by *japonica* rice with the *Wx* ^b^ allele, which has low AC and meets local preferences for a sticky and soft texture. Rice with extremely low AC, such as glutinous rice, has a soft texture and high stickiness, making it commonly used in desserts or festive foods in Asian regions. The ancestral allele *Wx* ^lv^ in wild rice diverged into modern cultivated alleles like *Wx* ^a^ and *Wx* ^b^ via single‐nucleotide substitutions, reflecting the response of artificial selection to regional demands (Zhang, Zhu, et al. 2020). Different *Wx* alleles influence regional adaptability by altering starch structure. The *Wx* ^b^ allele increases the proportion of short chains in amylopectin, enhancing rice viscoelasticity and making it more popular in the Japanese market (Cao et al. 2017). The *Wx* ^a^ allele reduces starch relative crystallinity and swelling power, making it suitable for producing boil‐resistant Basmati rice in India (Teng et al. 2016). By reducing AC to a moderate level (approximately 12%), the *Wx* ^mw^ allele improves eating quality while maintaining grain transparency. This trait is commonly used to improve the quality of some high‐yielding *japonica* rice varieties, such as the cultivation of elite varieties in the Nangeng series (Zhang et al. 2020).

#### 2.1.2 Amylopectin

Rice with the same amylose content differs in ECQ mainly due to amylopectin (AP) fine structure. AP's chain‐length distribution affects rice ECQ and starch physicochemical properties. AP branch chain length is correlated with starch gelatinisation temperature (GT): short‐chain AP content is negatively correlated with GT, long‐chain AP content is positively correlated. High long‐chain AP rice has firmer GC; high short‐chain AP rice has softer GC, and GC is negatively correlated with GT (Tian et al. 2009).

Granule‐bound starch synthase (GBSS) dominates amylose synthesis, while soluble starch synthases (SSs), starch branching enzymes (SBEs), and starch debranching enzymes (DBEs) mainly regulate amylopectin synthesis. SSs extend amylopectin branches (subtypes: SSI, SSII, SSIII, SSIV). SSI absence does not affect seed/starch granule size/morphology or endosperm starch crystallinity (other SSs compensate), but its absence/down‐regulation alters amylopectin chain‐length distribution (Fujita et al. 2006); *japonica SSIj* alleles bring shorter amylopectin chains and better ECQ than *indica SSIi* (Luo et al. 2015). *SSIIa* (*ALK*) controls starch GT and synthesizes AP medium chains (Degree of polymerization, DP13–25) (Gao et al. 2004); *ALK* mutants increase AP short chains (DP ≤ 12) and reduce GT (Zhang et al. 2011). At least four *ALK* alleles affect rice GT: *ALK* ^c^, *ALK* ^d^ (high GT); *ALK* ^a^, *ALK* ^b^ (low GT) (Chen et al. 2020a). Down‐regulating *OsSSIIb* reduces *Wx* and *OsSSIIa* expression, lowering amylopectin medium chains (DP13–24), increasing short chains (DP5–12), and improving rice cooking quality (Li et al. 2018). SSIII synthesizes long B‐chains (DP > 35), while SSIV initiates transient starch granules (Fujita et al. 2007). SSIVb elongates AP long chains, but SSIVa/b does not affect endosperm starch content/structure (Toyosawa et al. 2016). SSIIIa and SSIVb overlap in synthesising amylopectin branches (DP ≥ 42) (Toyosawa et al. 2016). *ss3b* and *ss3a‐ss3b* mutants increase amylopectin B1 chains (DP13–24), but only ss3a‐ss3b increases B3 chains (DP ≥ 37) (Huang et al. 2023). *OsSSIIIa* mutation raises rice endosperm amylose, resistant starch, lipids, amylose‐lipid complexes and gelatinisation temperature; *OsSSIIIb* mutation does not, showing functional redundancy (Huang et al. 2023).

SBE has five isoforms: SBEI, SBEIIa/b, SBEIII, SBEIV. It cleaves α‐1,4 glycosidic bonds (from SSS), transfers cleaved sugar chains to glucan C_6_, forming α‐1,6‐linked branches for amylopectin. SBEI acts on medium/long B‐chains; SBEII forms short A‐chains (6× more efficient than SBEI). SBEIIa makes DP6‐12 short chains, SBEIIb (rice endosperm‐specific) controls DP6‐7 short chains (Fujita et al. 2006). SBEIII/IV have short‐chain transfer/amylopectin catalytic activity; SBEIV may matter in late rice grain filling starch synthesis (Jiang et al. 2003). DBE includes isoamylase (ISA1/2/3) and pullulanase (PUL), hydrolyzing α‐1,6 glycosidic bonds. ISA ensures correct amylopectin synthesis: ISA1 forms homo/hetero‐oligomers (hetero‐oligomer dominates rice endosperm amylopectin biosynthesis, homo‐oligomer supplements) (Utsumi and Nakamura 2007). PUL mainly degrades branches linked by α‐1,6 glycosidic bonds and has a compensatory effect on ISA (Zeeman et al. 2010). DBE hydrolyzes incorrect amylopectin branch short chains; via disproportionating enzyme (DPE), long chains form for starch/α‐glucan phosphorylase (PHO/SP) (Takaha et al. 1998). Rice DPE (plastid DPE1, cytoplasmic DPE2) and PHO (stromal/L‐type PHO1, cytoplasmic/H‐type PHO2) each have two isoforms (Qin et al. 2016).

### 2.2 Protein——The Second Most Important Determinant of Rice CEQ

#### 2.2.1 The Relationship Between Rice Protein and CEQ

Rice protein is one of the important factors affecting its cooking and eating quality. Although there are currently inconsistent reports and differences regarding the relationship between protein and rice CEQ, most scholars believe that the protein content (PC) has a very significant negative correlation with the rice gel consistency, the starch breakdown value and peak viscosity, as well as the eating quality value of cooked rice, and has a very significant positive correlation with the setback value (Lou et al. 2023; Zhang et al. 2014). Increasing rice PC has a negative impact on its cooking and eating quality. The reason for this may be that the internal arrangement structure of rice with a high protein content is dense, and the spaces between starch granules in the endosperm are small, which in turn affects the gelatinisation of starch during the cooking process, resulting in an increase in the GT of starch and a decrease in the degree of doneness of cooked rice (Ding et al. 2012). Therefore, moderately reducing the content of storage proteins in the endosperm is one of the feasible strategies to improve rice CEQ.

#### 2.2.2 Key Genes for Grain Protein Content

Progress in research on rice grain protein content has been comprehensively reviewed in our previous review (Lou et al. 2023) and will not be reiterated herein; only the latest advances are supplemented. Recently, Cao et al. reported that the OsGATA7‐SMOS1 module regulates rice protein content and taste value by modulating the expression of the *OsGluA2*, *OsAAP6*, *RM1*, and *OsGluD* genes (Cao et al. 2025). Lou et al. demonstrated that *Ghd7* negatively regulates rice grain protein content while positively regulating amylose content and eating quality; in contrast, its antagonistic interacting factor, *OsNAC42*, exerts the opposite effects (Lou et al. 2025). All in all, we have summarised all currently identified regulators or potential regulators of grain protein content and mapped them to the chromosomes as illustrated in Figure 2.

### 2.3 Lipid——The Third Most Important Determinant of Rice CEQ

#### 2.3.1 The Relationship Between Rice Lipid and CEQ

Although lipids account for only 0.3%–0.6% of the components in polished rice, their impact on the eating quality of rice should not be underestimated. The eating quality of rice is an extremely complex trait, with a wide range of evaluation indicators. Among them, the taste value can intuitively reflect the palatability of cooked rice, while the analysis of RVA (Rapid Visco Analyzer) characteristic profiles simulates the rice cooking process to obtain various physicochemical properties of starch, serving as one of the effective means for evaluating the eating quality of rice. Cooked rice with high breakdown values and low setback values generally exhibits superior eating quality (Liu et al. 2011). Studies indicate that saturated fatty acid content is significantly negatively correlated with breakdown values but significantly positively correlated with setback values; conversely, unsaturated fatty acid content is significantly positively correlated with breakdown values and significantly negatively correlated with setback values (Xu et al. 2014). Additionally, fatty acids can form various complexes with starch, disrupting the internal helical structure of starch and making the internal structure of rice more compact, thereby reducing water absorption capacity. Such rice tends to have a chewier texture, distinct grains, and non‐stickiness after cooking. It is worth noting that unsaturated fatty acids are also the primary source of rice aroma during the formation of volatile flavour compounds in cooked rice.

#### 2.3.2 Genes Controlling Rice Lipid Metabolism

Although rice lipid has lower content and fewer related studies compared to the other two major endosperm storage substances—starch and protein—it is rich in unsaturated fatty acids of high quality. Therefore, identifying and cloning genes related to fatty acid content in rice is of significant scientific and practical importance.

SLL1 regulates global fatty acid desaturation, and OsFAD3 catalyses the conversion of linoleic acid to ALA (Shelley et al. 2013). *OsFAD2‐1* suppression via RNAi increases oleic acid content while decreasing linoleic and palmitic acid levels (Zaplin et al. 2013). Through GWAS and linkage analysis, 46 fatty acid‐related loci (including 26 genes) were identified, 16 of which were validated in RILs, and genes *PAL6*, *LIN6*, *MYR2*, and *ARA6* were cloned (Zhou et al. 2021). *FGC3* was identified through mGWAS and pGWAS and can be regulated by OsMADS6/OsMADS17, thereby affecting cutin monomer synthesis and grain yield (Yang et al. 2024). OsKCS11 regulates very‐long‐chain fatty acids and influences cytokinin metabolism, while OsWRI1a/OsWRI1b can upregulate genes related to glycolysis and fatty acid synthesis (Zhou, Pang, et al. 2025). Silencing of *OsLTPL36* impairs seed germination and reduces fatty acid content; suppression of *OLE16/OLE18* leads to oil body swelling and decreased triglyceride levels; and loss of *OsLPD1* function disrupts redox balance and reduces fatty acid accumulation (Chen et al. 2024).

### 2.4 Other Factors Influencing Rice CEQ

The final formation of rice CEQ is highly complex. Apart from the genes directly involved in the synthesis and metabolism of starch, protein, and lipid, other genetic factors—including those regulating aroma, chalkiness (or high‐temperature response), grain shape, sugar transport and metabolism, and NUE—also influence eating quality. They exert this influence either by indirectly regulating the composition, structure, or distribution of these three core endosperm components, or by altering the physicochemical properties (such as water absorption and texture development) during the cooking process. Notably, some of these genes even exhibit ‘pleiotropic regulation’ effects.

Of particular importance, the elite alleles of these indirect regulatory factors for eating quality share a key common feature: they all achieve the synergistic improvement of rice quality and yield by increasing starch content while decreasing protein content, a mechanism referred to as the ‘trade‐off between starch and protein’. The following sections elaborate on these categories of indirect eating quality factors respectively:

#### 2.4.1 Aroma

Rice cooking and eating quality depends on physical/sensory properties (stickiness, hardness, aroma). Key aromatic compounds are 2‐acetyl‐1‐pyrroline (2AP, main in fragrant rice like jasmine/basmati) and fatty acid‐derived volatiles (FAVs) (Li et al. 2024). 2AP forms when *BADH2* mutates (e.g., 8‐bp deletions, SNPs): non‐fragrant rice's active BADH2 converts 2AP precursor GABald to GABA; mutant *BADH2* lets GABald accumulate, forming 2AP via cyclisation (e.g., during cooking). *BADH2* has 19 major allelic variants (e.g., *badh2‐E2*, *badh2‐E7*) with distinct origins; evolution analysis shows fragrance genes first appeared in *japonica*, challenging the *indica* origin hypothesis (Bigyan et al. 2021). Li et al. (2024) found *OsWRKY19* (negatively regulates BADH2 to boost 2AP and agronomic traits) and *OsNAC021* (negatively regulates FAVs via lipoxygenase pathway; its knockout increases FAVs without yield loss) regulate aroma.

#### 2.4.2 Chalkiness

Rice chalkiness reduces grain transparency/appearance quality and impairs cooking/eating properties: loose starch in chalky regions causes uneven cooking water absorption, higher hardness, and lower viscoelasticity, worsening taste; high chalkiness often links to abnormal amylose content; Key genes affect chalkiness and storage substances: *Chalk5* overexpression raises chalkiness, amylose, and gel consistency, but lowers protein (Li et al. 2014); *WCR1* overexpression reduces the white‐core rate and protein (mainly glutelin), while increasing starch, amylose, and eating quality (Wu et al. 2022); *WBR7* knockout boosts starch, amylose, and protein (Shi et al. 2024); *chalk10* mutant increases chalkiness, loosens starch granules, and reduces starch/amylose, with CHALK10 interacting with SD1 to regulate gibberellin and chalkiness (Zhou et al. 2025); *swb9* mutant raises chalkiness but lowers starch/amylose and increases short‐chain amylopectin (Chen et al. 2025); *OsSPL14/IPA1* loss reduces starch/amylose but raises protein (Li et al. 2025); *LCG1* knockout worsens cooked rice luster/eating quality and alters viscosity, while introgressing Nipponbare's *LCG1* fragment reduces chalkiness and improves eating quality (Tu et al. 2024). In short, chalkiness forms with changes in rice storage substances, explaining why chalkiness genes affect eating quality.

#### 2.4.3 Grain Shape

Grain shape genes affect rice eating quality mainly by regulating chalkiness, storage substances, and grain properties. Slender grains (high length‐width ratio) usually have lower chalkiness—grain shape genes reduce chalkiness to enhance grain transparency and cooked rice uniformity/texture. Due to pleiotropy: *GS3/GW5/qSW5* variants alter starch granule density and pasting properties (Hao et al. 2023); *GL7/GW7/SLG7* promotes slender grains, lowering chalkiness and raising head rice rate/eating quality (Xia et al. 2025); *OsMADS1* mutations disrupt the expression of starch genes, impairing eating quality (Li et al. 2020); *GLW7.1*‐carrying lines have less chalkiness, higher amylose, and better eating quality (Liu et al. 2022). Slender grains absorb water uniformly when cooked (meeting quality standards) (Li et al. 2023); grain shape genes reduce milling breakage via regulating thickness to keep head rice (Yang et al. 2023). In short, grain shape genes improve eating quality by reducing chalkiness, optimising starch, and enhancing grain properties.

#### 2.4.4 Sugar Transport and Metabolism

Rice chalkiness (from filling defects) links to abnormal sugar metabolism/transport, worsening cooked rice texture and eating quality. *WBR7* (encodes SUS3) allelic variations affect chalkiness—hypofunctional alleles (e.g., Jin 23B) reduce chalkiness, improving quality (Shi et al. 2024). *qGT3* (encodes OsMADS1) regulates grain thickness, modulating *MST4* to affect filling‐stage sugar transport (Liu et al. 2025). *OsSUT/SWEET* families dominate rice sugar transport (apoplastic phloem loading); *OsSWEET1b* participates in glucose/galactose transport, influencing grain sugar/starch and eating quality (Shi et al. 2024). Carbohydrate transport efficiency determines grain plumpness/starch—filling defects raise chalkiness, while low transport disrupts amylose/amylopectin ratio, altering cooked rice viscoelasticity/hardness (Samonte et al. 2024). Thus, sugar transport genes are potential targets for improving rice eating quality.

### 2.5 The Key Regulatory Modules Coordinating Storage Protein and Starch Accumulation in Rice Seeds

**FIGURE 6:**
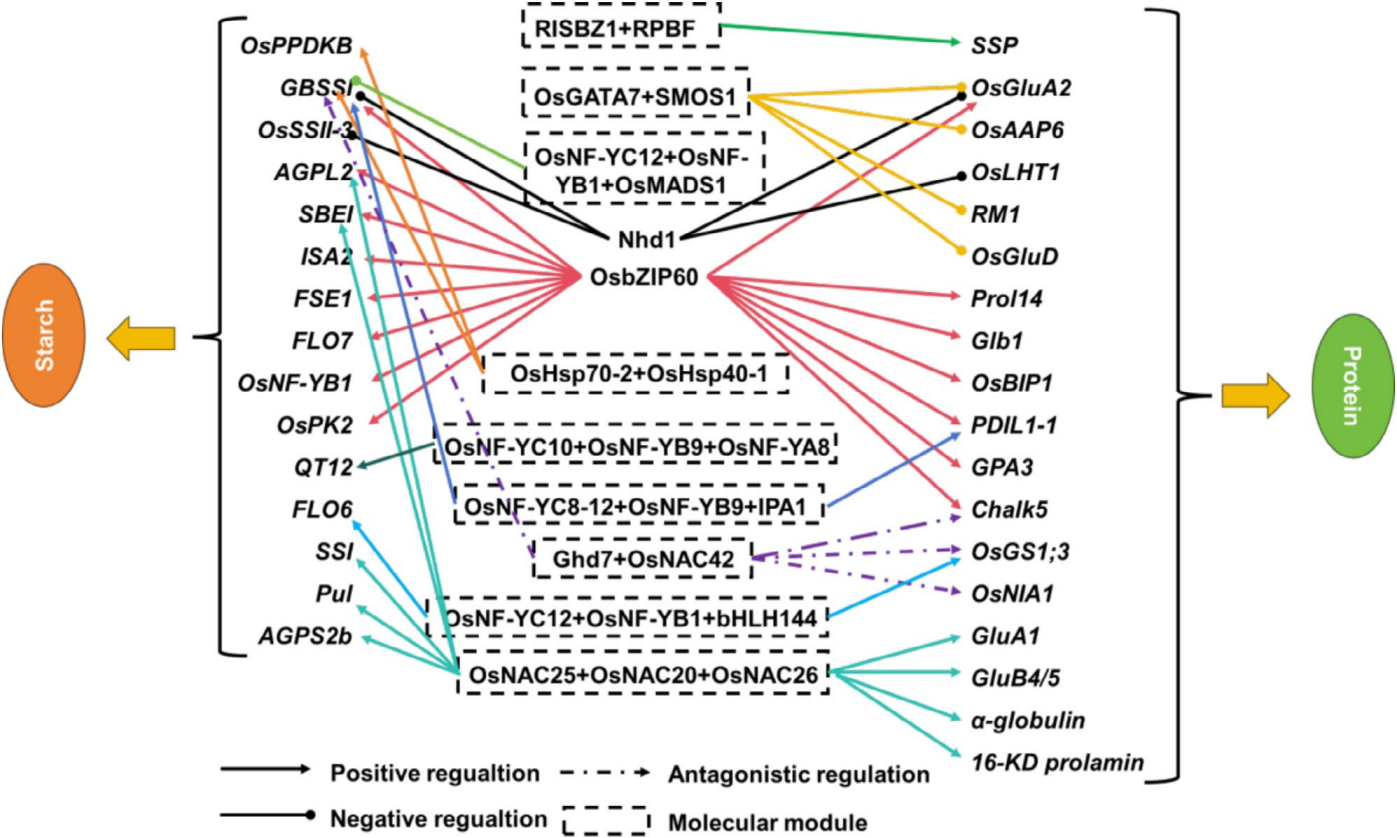
Key regulatory modules for storage protein and starch accumulation in rice grains, and their interaction relationships. Detailed information is described in the main text. A plus sign indicates the presence of interactions.

During endosperm development, the synthesis of seed storage proteins and starch proceeds concurrently, with both processes competing for resources and energy, often exhibiting a trade‐off relationship of reciprocal inhibition (Kavakli et al. 2000). Therefore, in a strict sense, it is impossible to completely decouple the biosynthesis and metabolism of these two storage substances. Floury endosperm mutants represent a classic example of this interdependency. Although most floury mutants were initially identified due to defects in starch synthesis, these starch‐deficient mutants typically exhibit concomitant changes in protein content. A comprehensive summary of this phenomenon was previously provided in our earlier work (Lou et al. 2023), and thus will not be repeated here. Here, we summarise and analyse the key regulatory modules coordinating storage protein and starch accumulation in rice seeds (Figure 6).

RISBZ1 and RPBF (maize O2/PBF1 orthologs) synergistically activate seed storage protein (SSP) genes via physical interaction; their downregulation reduces seed protein, starch, and lipids. RISBZ2‐5 (with RISBZ1) form heterodimers, and RISBZ2 binds *Wx* and α‐globulin motifs. OsbZIP60 activates SSP/starch genes, regulates ER protein processing (via *OsBIP1/PDIL1‐1*), and controls chalk‐related genes (Cao et al. 2022). OsNAC20/26 activate SSP/starch genes; single mutations have minor effects, but double mutations reduce starch/SSPs (functional redundancy) (Wang, Chen, et al. 2020). OsNAC25 (OsNAC20/26 upstream regulator) forms positive/negative loops with them to balance starch gene expression and accumulation. *qPC1* (encodes OsAAP6) boosts seed storage protein synthesis by transporting amino acids, and regulates starch genes (promotes GBSS/SS/DBE, inhibits BE) to increase amylose, decrease amylopectin (Peng et al. 2014). *Indica*‐specific *Chalk5* promotes chalk formation, upregulates 12 storage metabolism genes, causing abnormal amylose accumulation and lower protein, balancing starch‐protein (Li et al. 2014). NF‐YC12 interacts with NF‐YB1 to regulate *OsSUT1*, binds *FLO6/OsGS1;3* promoters, and with bHLH144 stabilises NF‐YB1 to activate *Wx* (Xiong et al. 2019). *Wx*, which regulates grain amylose synthesis, was found to also affect endosperm storage proteins (its 2.3 kb mRNA is negatively correlated with albumin) (Chen et al. 2018). *qGT3's OsMADS1* ^nyz^ activates *MST4* (enhances grain plumpness), suppresses *Wx* to reduce amylose/protein, increase total starch/amylopectin and eating quality (Liu et al. 2025). OsSPL14/IPA1 and OsNF‐YB9/YC8‐12 regulate the expression of *Wx*/*PDIL1‐1* in the form of a complex, thereby affecting the accumulation of starch and protein in the endosperm (Li, Guo, et al. 2025). Circadian regulator OsCCA1/OsLHY/Nhd1 binds *OsSSII‐3*, *Wx*, *OsGluA2*, *OsLHT1* to regulate rice storage substances and eating quality (Zhang et al. 2023). OsGATA7‐SMOS1 modulates protein biosynthesis genes (e.g., *OsGluA2*) to affect eating quality, and also alters endosperm starch, coordinating storage substance accumulation (Cao et al. 2025). *QT12* (encodes Sec61 β subunit) affects storage balance: high expression reduces protein/increases amylose (higher chalkiness); low expression maintains balance/enhances heat tolerance. Upstream NF‐Y complex participates in temperature response and storage regulation (Li et al. 2025). OsHsp70‐2/OsHsp40‐1 form a chaperone module, stabilising OsGBSSI/OsPPDKB to promote starch synthesis; their mutants reduce starch and protein, critical for both accumulations (Lu, Jiao, et al. 2024). Ghd7 (reduces protein, boosts amylose/eating quality) and OsNAC42 (opposite functions) form a heterodimer to antagonistically regulate starch/protein genes, balancing quality and yield (Lou et al. 2025).

Analysis of above findings shows the OsNF‐Ys complex is pivotal for regulating rice seed storage protein and starch accumulation. Factors like OsbZIP60, OsMADS1, QT12, and Ghd7 interact with it when modulating endosperm storage‐related genes. Characterising its upstream/downstream interactors helps dissect rice storage biosynthesis networks.

In another study, we previously summarised the nitrogen‐use efficiency‐related genes reported in rice that may influence grain protein content (Lou et al. 2023). Over time, increasing evidence has validated the accuracy of this inference. To date, *Nhd1*, *OsMADS1*, *OsSPL14*, *OsNAC42*, and *Ghd7* have all been confirmed to participate in regulating grain protein content and are recognised as key regulatory modules governing the accumulation of storage proteins and starch in rice seeds.

## 3 Breeding Strategies for Rice CEQ

**FIGURE 7:**
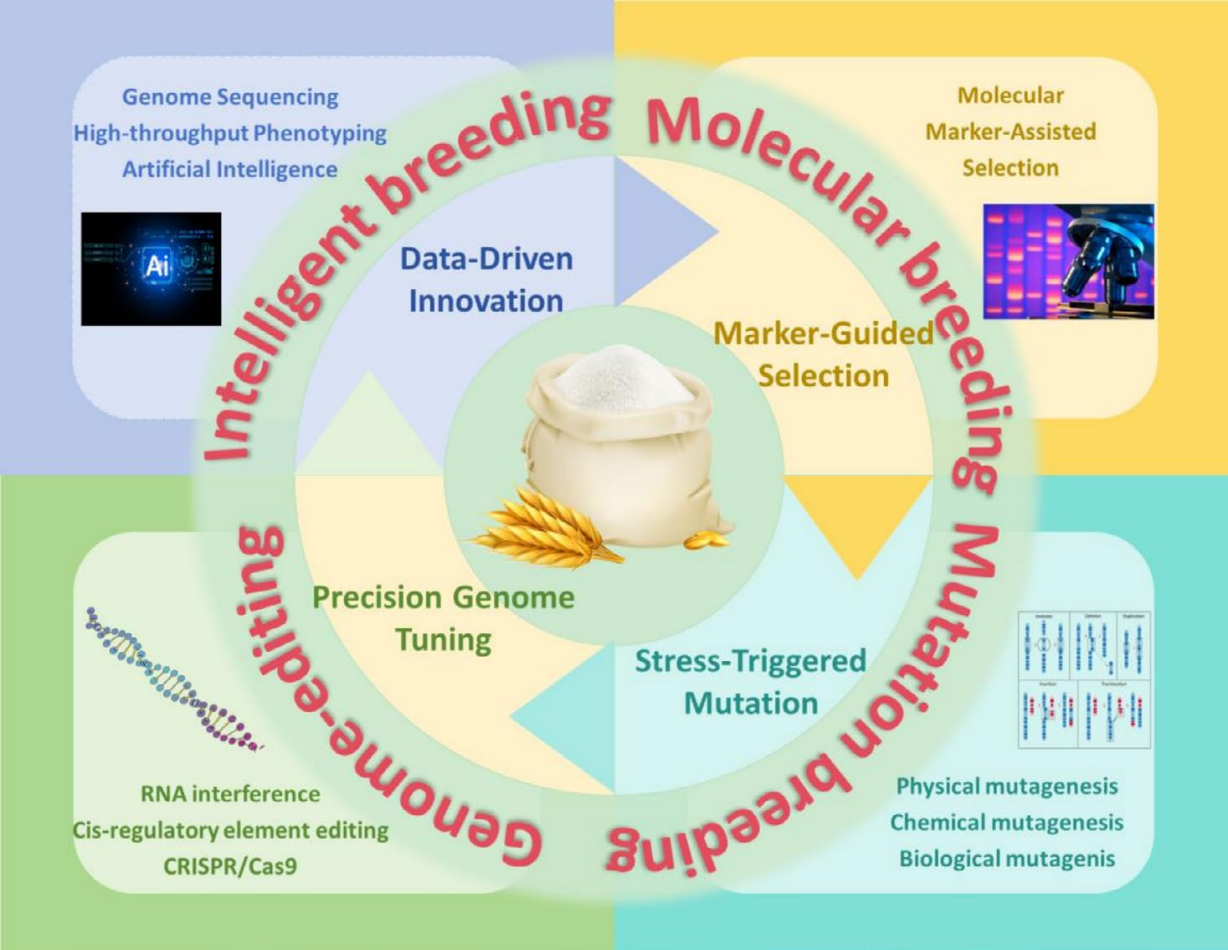
Molecular breeding strategies for improving rice CEQ. This diagram adopts a circular layout, with grains at the center (symbolising the breeding objectives). It divides and presents four major molecular breeding strategies, each corresponding to distinct technical pathways. This clearly demonstrates how modern molecular breeding technologies synergistically enable the genetic improvement of crops.

Variety improvement and breeding are pivotal agricultural practices that facilitate the systematic selection and utilisation of beneficial genetic resources within existing cultivars. However, this endeavour is inherently time‐consuming, and conventional breeding strategies often suffer from low precision, which in turn limits their ability to achieve optimal outcomes. Therefore, integrating the improvement of rice CEQ with diverse breeding strategies can enhance the efficiency, target specificity, and overall effectiveness of this process. Consequently, this review systematically summarises the prevalent and promising breeding strategies (Figure 7), as well as the underlying conceptual frameworks, applicable to the improvement of cooking and eating quality in current and future agricultural practices.

### 3.1 Molecular Breeding Strategy

Directional regulation and utilisation of key genes governing rice CEQ represent the most direct and effective improvement strategy. To date, breeders have achieved notable progress through molecular breeding approaches and rational improvement strategies for targeted modification. Leveraging *Wx* ^mp^, an allele controlling low amylose content and superior eating quality, researchers have successfully developed a series of elite *japonica* rice varieties, including ‘Nanjing 46’, ‘Nanjing 5055’, and ‘Nanjing 9108’ (Sreenivasulu et al. 2022). By introgressing the *Wx* ^mp^ allele (conferring excellent taste quality) and blast‐resistant alleles into the genetic backgrounds of two high‐yielding cultivars, two elite lines—XY99 and JXY1—were developed, exhibiting superior palatability, high yield, and broad‐spectrum blast resistance (Xiao et al. 2021). Additionally, introducing the high‐expression *OsAAP6* allele from Zhenshan 97 into the *japonica* variety ‘Nanyangzhan’ significantly enhanced rice nutritional quality (Peng et al. 2014). Similarly, introgression of the *ALK* allele from 9311 into the *japonica* cultivar ‘Nipponbare’ markedly improved rice CEQ (Chen et al. 2020b).

### 3.2 Mutation Breeding Strategy

The availability of rare beneficial alleles in nature severely limits human selection of target traits in traditional crops. To overcome these limitations, mutation breeding techniques have been developed to introduce non‐naturally occurring alleles through physical, chemical, and biological means of random mutagenesis. For example, Lin et al. (2024) identified a new *Wx* allele named *Wx* ^ela^ from an ethyl methanesulfonate (EMS)‐induced mutant of Wuyunjing 8. This allele causes an amino acid substitution (T506I) in the GT1 domain of granule‐bound starch synthase I (GBSSI), reduces GBSSI activity, and can synthesize extremely low amylose to improve the eating and cooking quality of rice. However, this method has significant limitations and is difficult to play a greater role in practical production until these defects are completely overcome. First, after initial mutagenesis, a large population must be screened to identify mutants with ideal grain quality characteristics. Second, due to the unpredictability of mutations, after consuming substantial human and material resources, the desired mutant traits are not always produced; most often, the mutations generated are harmful.

### 3.3 Genome‐Editing Breeding Strategy

Genetic engineering and plant transformation technologies improve crop CEQ by introducing foreign genes or silencing endogenous ones. RNA interference (RNAi) suppresses quality‐related genes (*Wx*, *SSI*, *SSII*‐2, *ALK*, *OsFAD2*‐1), producing transgenic rice with better CEQ (Sreenivasulu et al. 2022); however, transgenic crops face obstacles like safety controversies and regulatory complexities. Genome‐edited crops (no foreign genes) are more accepted: CRISPR/Cas9 edits *Wx* (promoter, exons, introns) to alter amylose content and CEQ (Huang et al. 2020; Xu et al. 2020; Zeng et al. 2020). Wang et al. (2025) edit 14 amylopectin synthesis genes, increasing resistant starch but reducing CEQ; Yang et al. (2022) and Chen et al. (2022) knock out glutelin genes, lowering protein and boosting taste; Wang et al. (2024) edit *OsNF‐YC4* promoter (raises protein, lowers starch) and knock out *OsAAP6/OsAAP1* (reduces protein, improves CEQ) (Wang, Yang, et al. 2020); *FAD2‐1* knockout creates high‐unsaturated fatty acid rice (Abe et al. 2018).

Rice CEQ (softness, extensibility, stickiness) is regulated by *Wx* and minor genes; synergistic multi‐gene regulation (e.g., *Wx* and *SSII*) enhances CEQ (Huang et al. 2021). Editing cis‐regulatory elements fine‐tunes gene expression: Lu, Zhang, et al. (2024) edit *Wx*'s uORF6 to upregulate *Wx*, setting amylose to ~1.5% (semi‐*waxy* japonica), and inserting uORF6 copies to improve high‐amylose rice (Lu, Zhang, et al. 2024); Yang et al. (2025) edit *OsGLW7/OsGW7* uORFs, lowering amylose, increasing gel consistency, and softening texture (Yang et al. 2025).

### 3.4 Modern Intelligent Breeding Strategy

The modern intelligent breeding strategy is a precision breeding model centered on improving the cooking and rice CEQ, integrating multi‐dimensional technologies. Specifically, it involves constructing a ‘genotype–phenotype‐CEQ’ association database via platforms such as RiceNavi (Wei et al. 2021), and leveraging AI‐driven genomic selection (GS) models to predict the eating quality potential of unplanted germplasm at the seedling stage, thereby enabling efficient screening of parental lines carrying elite genes (e.g., *Wx* and *ALK*). It achieves precise improvement of key quality‐related genes and creation of customised alleles through marker‐assisted backcrossing (MABC), marker‐assisted gene pyramiding (MAGP), and CRISPR‐based gene editing; meanwhile, AI can assist in optimising molecular marker design and predicting editing outcomes. Relying on AI‐empowered high‐throughput phenotyping (e.g., UAV‐based hyperspectral sensing modelling and data analysis of automated laboratory detection platforms), it overcomes the limitations of traditional detection methods, realising large‐scale, non‐destructive preliminary quality screening and efficient analysis. Based on the AI‐constructed dual‐target (quality and yield) GS model and the gene balance hypothesis, it enables intelligent design of hybrid combinations, predicts the eating quality and yield performance of F_1_ generations, and screens for combinations with ‘superior quality without yield reduction’. Additionally, by integrating the pleiotropy of the *Wx* gene and the effects of multi‐gene networks, it facilitates the directional development of high‐quality rice varieties tailored to regional preferences.

With the deepening of our understanding of the pleiotropy of the *Wx* gene, the genetic improvement of rice eating quality needs to be reinterpreted from a new perspective. As the most critical determinant of eating quality, the *Wx* gene primarily exerts its regulatory effects through the contents of amylopectin, glutelin, and lipids. Although various *Wx* genotypes have been well characterised so far, this does not equate to a complete understanding of the mechanisms underlying eating quality formation. Notably, significant variations in eating quality scores may occur among different rice varieties carrying the same *Wx* allele. This phenomenon is mainly attributed to the combined effects of amylopectin, glutelin, and lipids, which are regulated by a complex multi‐gene network. Therefore, in the practice of improving rice eating quality, the following approach should be adopted: first, determine the target *Wx* genotype based on regional preferences for rice texture; second, perform targeted regulation of amylopectin and glutelin contents; ultimately, achieve the goal of breeding rice varieties with superior eating quality through rational breeding strategies.

## 4 Conclusion

The improvement of rice CEQ should be carried out based on the principle of ‘taking genetic regulation as the core, technological innovation as the support, and regional demand as the guide’. As the central hub for CEQ regulation, the allelic genotype of the *Wx* gene should first be matched with the taste preferences of the local population. Subsequently, by targeted regulation of amylopectin synthesis genes (e.g., *SSIIa*, *SBEIIb*) and glutelin‐related genes (e.g., *OsAAP6*), the starch‐protein ratio can be optimised, laying a foundation for high‐quality taste. Meanwhile, it is necessary to synergistically utilise genes related to grain shape (e.g., *GS3*, *GLW7.1*), chalkiness (e.g., *Chalk5*, *WBR7*), sugar transport (e.g., *OsSWEET1b*), and nitrogen use efficiency (e.g., *Ghd7*) to address the trade‐off dilemma between ‘quality and yield’. At the technical level, an efficient and precise breeding system has been established, which integrates CRISPR/Cas9‐mediated targeted editing, marker‐assisted multi‐gene pyramiding, as well as artificial intelligence (AI)‐driven genomic selection and high‐throughput phenotyping. In the future, following the strategy of ‘matching regional *Wx* genotype preferences → regulating key substance contents → synergistic breeding with multiple technologies’, rice varieties with excellent CEQ, high yield, and stress tolerance can be further developed, providing support for global food security and the upgrading of consumer demands.

## 5 Materials and Methods

### 5.1 Plant Materials and Growth Conditions

The construction details of a series of transgenic lines with the Zhennuo genetic background carrying distinct *Wx* alleles have been elaborated in prior publications (Xia et al. 2022). These materials were cultivated at the Huazhong Agricultural University Farm (30.49° N, 114.36° E) during the 2023 rice growing season. All genotypes were sown on June 20th, with seedlings transplanted from the seedbed to the field exactly 1 month later. The planting density was set at 16.5 cm within rows and 26 cm between rows, following standard agronomic practices recommended for the region. Each line was planted in three biological replicates, with each replicate comprising 36 plants arranged in 3 rows of 12 plants. To mitigate edge effects, only plants from inner rows were harvested at maturity. Harvested grains were air‐dried, stored at ambient temperature, and subsequently subjected to trait measurements.

### 5.2 Measurements of Cooking and Eating Qualities

For CEQ analysis, mature seeds were dried in an oven at 37°C for 24 h prior to dehulling and milling. Milled rice flour was used for determining amylose content, total starch, and four storage proteins. Mature seeds were first dehulled using a Nong'ao brand hulling machine (model NA.2058), followed by milling into polished rice with a LOOBO small‐scale rice mill (model LB‐JNM‐III). The polished rice was then ground into flour and sieved through a 200‐mesh sieve. Amylose content was measured using the iodine‐potassium iodide (I_2_‐KI) method with 0.01 g of rice flour (Bao et al. 2006). Procedures for determining the four storage proteins were adapted from previous studies (Chen et al. 2018). Fatty acid analysis was performed using brown rice and analyzed by gas chromatography, with specific details described in previous reports (Zhou, Xia, Li, et al. 2021). The determination of taste value was conducted using polished rice, and the specific methodology has been meticulously described in previous studies (Xia, Wang, et al. 2022).

### 5.3 Statistical Analysis

Data analysis was processed and refined in Microsoft Office Excel 2021, while one‐way analysis of variance (one‐way ANOVA) and Duncan's multiple comparisons were performed using IBM SPSS Statistics software (Version 22.0).

## Author Contributions

Y. He and G. Gao designed the experiments. G. Lou analyzed the data and wrote the manuscript. P. Fu completed the phenotypic evaluation and preliminary data analysis of genetic materials with different *Wx* allelic genotypes. H. Zhou, D. Xia and Y. Li provided guidance in the relevant phenotypic determination. H. Gao, M. Li, H. Shi and R. Liu provided assistance in the relevant sample collection, processing, and phenotypic evaluation. Y. He and Y. Wang provided reference suggestions during the manuscript revision. All of the authors read and approved the manuscript.

## Conflicts of Interest

The authors declare no conflicts of interest.

## Supporting information


**Table S1:** Effects of different *Wx* allelic genotypes on various rice quality traits.
**Table S2:** Key information on confirmed and potential CEQ genes.

## Data Availability

The data that supports the findings of this study is available in Table S1 and Table S2 of this article.
